# The Eyes Test as a Measure of Individual Differences: How much of the Variance Reflects Verbal IQ?

**DOI:** 10.3389/fpsyg.2012.00220

**Published:** 2012-07-05

**Authors:** Eric Peterson, Stephanie F. Miller

**Affiliations:** ^1^School of Psychological Sciences, University of Northern ColoradoGreeley, CO, USA; ^2^Department of School Psychology, University of Northern ColoradoGreeley, CO, USA

**Keywords:** theory of mind, RMET, face processing, vocabulary, mentalizing, social perception

## Abstract

Developed by Baron-Cohen et al. (1997, 2001), the Reading the Mind in the Eyes Test requires individuals to determine mental states from photos of pairs of eyes. Used in over 250 studies, it has been conceptualized as an advanced theory of mind test that is relatively free of general cognitive abilities. Given the sensitivity of the instrument, many studies with healthy adult samples have used this instrument as a measure of individual differences in social-perceptual processes that contribute to theory of mind and overall phenotype. We administered the two-subtest Wechsler Abbreviated Scale of Intelligence, a face-processing task (Cambridge Face Memory Test), and the Eyes Test to 42 college students. Surprisingly, verbal IQ contributed significantly to the variance in Eyes Test performance while the face perception measure did not. These findings have both practical and theoretical ramifications for interpreting Eyes Test results in normative adult samples.

## Introduction

The relative ease or difficulty an individual experiences in understanding others influences his or her outcome across a range of settings (e.g., career choice, relationship success). Toward the goal of understanding individual differences among high-functioning adults in mental state understanding, a wide array of instruments have been developed. By design, some measures (e.g., Strange Stories Task: Happe, 1994; and the *Faux Pas* task: Baron-Cohen et al., 1999) involve making explicit inferences about the contents of another’s thoughts communicated linguistically (e.g., sentences of dialog between characters). Other instruments examine implicit social-perceptual processes like judging emotion or mental state information from non-verbal channels (e.g., facial display, voice). A number of researchers have argued that our full “mentalizing” ability rests on the integration of such fast and automatic implicit processes with more cognitively mediated explicit processes (Sabbagh, 2004; Frith and Frith, 2008; Apperly and Butterfill, 2009). Among the many tests used to study individual differences among high-functioning adults in mentalizing, the Reading the Mind in the Eyes Test (henceforth, Eyes Test) has emerged as a standard evidenced by its use in more than 250 studies, translation into several languages, and adaptations for many different research contexts (for reviews, see Johnston et al., 2008; Adams et al., 2009; Hallerback et al., 2009). Baron-Cohen et al. (2001) describe the task as involving “unconscious, rapid, and automatic” processes, consistent with an implicit task, relatively free from general cognitive ability. Despite its widespread use, the instrument has been minimally explored and the degree to which performance relies on implicit social-cognitive processes free from more general cognitive ability remains unknown.

The Eyes Test involves examining pairs of eyes (cut out from the face) and making a forced choice among descriptors to identify the mental state or emotion. Although it was first introduced in autism research (Baron-Cohen et al., 1997), the Eyes Test’s potential for studying individual differences among normally developing individuals was quickly established. First, in a later broad autism phenotype study, parents of children with autism spectrum disorders performed more poorly than comparison participants (Baron-Cohen and Hammer, 1997). In a series of subsequent studies aimed at examining individual differences in mentalizing among healthy adults, Baron-Cohen and colleagues demonstrated that the Eyes Test, in conjunction with other measures, discriminates between individuals with a propensity for humanities from individuals with a physical sciences orientation (Billington et al., 2007). To date, many researchers have used this instrument to probe for individual differences in theory of mind ability and personality (e.g., Dziobek et al., 2005; Carroll and Yung, 2006; Mar et al., 2006; Declerck and Bogaert, 2008; Ferguson and Austin, 2010; Lee et al., 2010; Sylwester et al., 2012).

In the current study, we explored the Eyes Test in conjunction with several tasks. Our choice of tasks was motivated by two general questions. Our primary question concerned the degree to which the Eyes Test correlated with IQ relative to a more basic face-processing task. Of course, given that the task involves choosing verbal descriptors it must certainly involve verbal ability. However, we presume that its strength as a social-cognitive index depends, in part, on whether variance is driven by the social-perceptual ability to glean mental state information from the eyes rather than a domain-general cognitive ability. To address our first question, we examined the relationship of two subtests of the Wechsler Abbreviated Scales of Intelligence (Vocabulary and Matrix Reasoning) and the Cambridge Face Memory Test (CFMT, Duchaine and Nakayama, 2006) to Eyes Test performance. The CFMT was chosen as a basic measure of face processing because, like the Eyes Test, it relies on a quick visual perception of the facial region. The CFMT was designed to be sensitive to the full spectrum of individual differences in face processing (i.e., from severely impaired to exceptionally skilled) and its validity has been demonstrated across a wide functional range (Duchaine and Nakayama, 2006).

Our secondary question reflects the contribution of the autism literature to our understanding of typical social cognition. The importance of the relevance of autism for understanding individual differences in typical social-cognitive development has received strong support from recent genetically sensitive studies (Constantino and Todd, 2003; Ronald et al., 2006). Collectively, these recent studies demonstrate that subclinical autism traits are normally distributed in the population. Thus, for example, one may possess a subset of the genetic risk factors for autism and express some specific autism-like traits (e.g., difficulty reading face emotion, difficulty with some aspects of language) without meeting criteria for diagnosis. We included measures of two language component skills that have been associated with genetic vulnerability to autism. Specifically, first-degree relatives of individuals with autism have more difficulty with phonological processing (Schmidt et al., 2008) and the pragmatics of language (Piven et al., 1997; Losh and Piven, 2007) compared to family members of non-autistic control participants. Thus, we selected two additional tasks to measure phonological memory and the appreciation of figurative language (i.e., understanding idiom and metaphor). The figurative language measure was of particular interest given that proficiency in the pragmatics of language requires an appreciation of the speaker’s intention and the social context. In other disorders besides autism, a relationship between pragmatic language ability and social understanding has been demonstrated (e.g., schizophrenia: Gavilan and Garcia-Albea, 2011; bipolar disorder: McClure et al., 2005). In this more exploratory aspect of our study, we reasoned that performance on one or both of these measures may account for some of the variance captured by verbal IQ. Alternatively, these additional measures may relate to Eyes Test performance independently from verbal IQ.

## Materials and Methods

### Participants

We recruited 45 college students from a medium-sized university. Participation involved two visits. Three participants did not return for a second visit, yielding a sample of 42 (23 males; Mean age = 19, Range = 17-34). Participants were excluded from the study if English was not their first language. All participants provided informed consent and received course credit. Our study was approved by our institution’s review board.

### Constructs and instrumentation

#### Understanding mental states based on examining the eye region

The revised version of the Eyes Test (Baron-Cohen et al., 2001) consists of 36 black-and-white photographs of the eye region. In each trial, participants are instructed to choose among four descriptors based on what the person in the photograph was thinking or feeling. In an earlier psychometric analysis of the Eyes Test including a test-retest reliability study, we identified 7 items that reduced the overall alpha in each of the two administrations (Peterson et al., in preparation). For the current study, these 7 items were eliminated so the test was administered with 29 items. Each item was accompanied by the same four response options included in the original Eyes Test. Participants were provided a glossary of definitions for all of the response choices. For the purpose of analysis, individual scores on the Eyes Test consist of the total number of correct responses.

#### Face recognition

The CFMT (Duchaine and Nakayama, 2006) is a computerized test consisting of a total of 72 trials, divided into three separate, progressively difficult stages in which participants are asked to recognize target faces among several distracter faces. The CFMT score is computed by summing the total of number of correct responses across all three stages.

#### Verbal and performance IQ

The Wechsler Abbreviated Scale of Intelligence (WASI) is a norm-referenced measure of intelligence that has well-established reliability and validity (Wechsler, 1999). We administered the two-subtest WASI (Vocabulary and Matrix Reasoning). In prior research, these two subtests have exhibited high factor loadings on a general intelligence factor (Kaufmann, 1994) and were therefore considered an adequate measure of overall intelligence. For each subtest raw scores were converted to age-corrected *T*-scores. Full-scale IQ was derived by summing the *T*-scores of the two subtests and converting the summed score to a standard score.

#### Phonological memory

The Non-word Repetition subtest of the Comprehensive Test of Phonological Processing (CTOPP, Wagner et al., 1999) consists of 18 items of pronounceable non-words. Participants listen to an audio recording of pseudowords of increasing difficulty and repeat the non-word aloud immediately after hearing it. The examiner judged each item as correct or incorrect on line. Raw scores were converted to age-correlated scaled scores.

#### Figurative language

The Figurative Language subtest of the Test of Language Competence (TLC, Wigg and Secord, 1989) was used to assess the ability to understand meaning intended in sentences involving relatively abstract language. On this 12-item task, participants are told about a situation and provided with a figurative expression (e.g., “He is high man on the totem pole”). They are first asked to describe the meaning of the expression in their own words. Then they are read and shown four response statements from which they choose the most equivalent alternative expression (e.g., “He is top dog”). Raw scores were converted to age-corrected scaled scores.

### Procedure

This study was part of a larger investigation of the Eyes Test. Data were collected across two testing sessions. In the initial session, participants completed the Eyes Test and CFMT. In the second session, participants completed the two-subtest WASI, CTOPP Non-word Repetition subtest, and TLC Figurative Language subtest.

### Data analysis

SPSS 18.0 statistical software was used to conduct all analyses. Distributional properties of our variables were examined and all were normally distributed. We examined the Pearson correlations among variables and a multiple regression analysis identified predictors accounting for unique variance in Eyes Test performance. An alpha value of 0.05 was used to establish the threshold for significance.

## Results

Table 1 provides the means and standard deviations for each task and Table 2 provides the Pearson correlations between the Eyes Test and scores on the WASI, CFMT, and CTOPP. Importantly, the mean IQ estimate for our sample of 42 college students (105) was close to national norms. While the Matrix Reasoning subtest did not significantly correlate with Eyes Test performance, the Vocabulary subtest did (*r* = 0.49, *p* = 0.001, a large effect size). Performance in the CFMT, our face-processing measure, was not associated with performance on either WASI subtest. There was no association between performance on the CFMT and the Eyes Test.

**Table 1:** **Descriptive data**.

Instrument	Means (SD)
Eyes test^1^	21.26 (2.85)
CFMT^1^	54.80 (7.98)
WASI vocabulary^2^	51.40 (7.82)
WASI matrix reasoning^2^	54.21 (6.29)
WASI full-scale IQ^3^	104.79 (9.92)
CTOPP non-word repetition^4^	9.19 (1.84)
TLC figurative subtest language^4^	8.69 (2.40)

*SD, standard deviation; CFMT, Cambridge Face Memory Test; WASI, Wechsler Abbreviated Scale of Intelligence; CTOPP, Comprehensive Test of Phonological Processing; TLC, Test of Language Competence*.

*^1^Raw score*.

*^2^T score with a population mean = 50, SD = 10*.

*^3^Standard score with a population mean = 100, SD = 15*.

*^4^Scaled score with a population mean = 10, SD = 3*.

**Table 2:** **Pearson correlations**.

	Eyes test	WASI vocabulary	WASI matrix reasoning	CTOPP non-word repetition	TLC FL	CFMT
Eyes test						
WASI vocabulary	**0.49**					
WASI matrix reasoning	0.18	0.29				
CTOPP non-word repetition	−0.05	−0.15	0.03			
TLC figurative language	**0.43**	**0.53**	0.26	−0.12		
CFMT	0.01	0.01	0.21	−0.17	0.22	

*Bolded correlations are significant at the 0.05 alpha level (two-tailed)*.

Our secondary question concerned whether two linguistic skills (phonological memory and appreciation of figurative language) contribute independently from verbal IQ to Eyes Test performance. The figurative language test but not the test of phonological processing related to individual Eyes Test scores. A follow-up regression analysis was conducted in which both of the verbal measures that had been significant in the bivariate analyses (verbal IQ, figurative language) were used to predict Eyes Test score. Total *R*^2^ was 0.28 (*p* = 0.002). Verbal IQ explained significant unique variance in Eyes Test performance (β = 0.36, *p* = 0.028), but figurative language did not (β = 0.24, *p* > 0.1).

## Discussion

The central finding of our study was that performance on the Eyes Test correlated to a surprising degree with verbal IQ and not with a more basic measure of face processing. We believe this finding has practical importance for considering the value of the Eyes Test as an instrument for studying individual differences in social cognition among adults. Further, we believe our results raise a question about the degree to which performance differences in this instrument reflect a relatively implicit mechanism.

Across the past decade or so, research emerging from a range of sub-disciplines has highlighted the importance of understanding the mechanisms supporting individual differences among adults in the ability to understand other’s mental states. A central premise of this effort is that our full mentalizing ability rests on a range of both explicit and implicit processes. Apperly and Butterfill (2009) have posited a dual route framework with one route characterized by speed and efficiency at the cost of flexibility, and another route that is slower, more cognitively demanding, and supported by domain-general cognitive processes like executive control. In the case of the Eyes Test, it is reasonable to speculate that implicit processes may be particularly important for task performance. One must examine a pair of eyes and make a relatively automatic judgment to choose the best mental state descriptor (the eyes look “skeptical” rather than “indifferent,” “embarrassed,” or “dispirited”). However, in no trial does performance require an explicit meta-representation, that is, a representation of another’s thought process (e.g., what exactly the person is skeptical about). Of course, it is important to differentiate between the minimal demands necessary for success as opposed to the important and ambiguous question as to what the participant thinks when examining the pair of eyes.

Presumably, individual differences on an implicit social-perceptual task should be minimally influenced by participant differences in verbal IQ. However, our results show that in a college sample with a mean IQ close to national norms, verbal IQ alone accounts for almost 25% of the variance in Eyes Test performance. In the current study, verbal IQ was estimated using a measure of expressive vocabulary that taps basic vocabulary knowledge as well as other expressive language skills. Following the original Eyes Test instructions, we provided a list of definitions for all the descriptors and we encouraged participants to consult the list whenever they felt uncertain about the meaning of a word. Nevertheless, it may be that the relation between verbal IQ and Eyes Test performance is driven primarily by individual differences in vocabulary knowledge. However, it seems more likely that other cognitive processes contribute substantially to performance differences. Reasonable candidates include verbal-reasoning and verbal working memory.

In order to obtain a more refined understanding of the cognitive processes contributing to the overlap between the Eyes Test and the Wechsler Vocabulary subtest, a future study should include both traditionally explicit and implicit mental state reading tasks as well as measures of vocabulary knowledge, verbal-reasoning, and verbal working memory. By design, mentalizing tasks, like the Strange Stories test and the *Faux Pas* test involve rich linguistic processing in order to extract explicit mental state information conveyed by sentences. The standard interpretation of the Eyes Test would predict a relatively greater verbal IQ contribution to these tests. However, our own results showing a surprisingly high correlation between the Eyes Test and verbal IQ cast some doubt on this prediction. In a recent meta-analytic investigation (Kirkland et al., 2012), we examined studies in which the Eyes Test and either the Strange Stories and/or the *Faux Pas* test were administered. Our results demonstrated a moderate correlation (*r* = 0.29) between performance on the Eyes Test and each of these measures. Inclusion of face emotion reading tasks like the Diagnostic Analysis of Non-verbal Behavior (DANVA) tasks that minimally involve language would provide an important opportunity to compare the relative contribution of general intelligence processes in each category of task.

Importantly, such a future study should include cognitive measures intended to explore more precisely the role of verbal ability. First, to eliminate the unlikely possibility that vocabulary knowledge alone contributes significantly, a vocabulary test could be included that specifically targets word meanings from the Eyes Test items. While the instrument includes a set of definitions and participants are encouraged to make use of it, it is unclear to what degree this intervention reduces the variance attributable to vocabulary knowledge. Second, it would be important to include other measures of verbal ability (e.g., Wechsler Similarities subtest) in order to examine verbal-reasoning ability as well as a measure of verbal working memory. It is well known that explicit tasks like the Strange Stories test involve executive control, in particular, working memory. It is possible that both the Eyes Test and the Strange Stories test (which our meta-analysis show to be moderately correlated) share an underlying component of general intelligence that may be common to tasks involving mental state reasoning. However, in our own single study, the correlation between the Eyes Test and the Vocabulary subtest was notably higher than the correlation with either the Strange Stories or the *Faux Pas* test (0.49 versus 0.29). Indeed, given that the standard deviation of our sample’s mean IQ score was smaller than that of the population, a restricted range correction (Thorndike, 1947) suggests *r* = 0.64 is a better estimate of the real effect. Of course, the effect size from the meta-analysis is much more likely to be free of sampling error than that of our single study.

In our study, more than 70% of the variance in Eyes Task performance remained unaccounted for. A central goal of our study was to ask whether some of the variance would be explained by a basic measure of face processing which was presumed not to overlap with verbal IQ. The CFMT was originally developed as a standardized measure of face recognition. In an initial study of the CFMT (Duchaine and Nakayama, 2006), the task yielded a wide range of scores that did not cluster near the floor or ceiling, suggesting that it was sensitive to the full spectrum of face recognition ability from severely impaired to significantly better than average (i.e., “super recognizers, ” Russell et al., 2009). Consistent with the conception of this task as a very basic face-processing measure, it did not show any correlation with verbal or performance IQ in the current study.

Considering the lack of any relationship between the Eyes Test and the CFMT in our sample, it is reasonable to question the lack of an explicit social emotional judgment in the CFMT as compared to the Eyes Test. However, given the profound social significance of the human face, even a very basic face-processing task could be expected to relate to broader social cognition. Indeed, one recent study examined the degree to which individual performance in face recognition for neutral faces correlated with performance in either the Eyes Test or the DANVA facial expression scale, an instrument that involves reading face emotion (Franklin and Adams, 2010). As predicted, participants who showed better memory for neutral faces performed better on face emotion reading. However, as in the current study, Franklin and Adams obtained no correlation between basic face recognition memory and Eyes Test performance. The authors did obtain a correlation between the Eyes Test and face emotion reading in spite of the lack of a relationship between the Eyes Test and the more basic face-processing task. Presumably, performance on the Eyes Test and the DANVA involved a common underlying ability that does not seem to relate to face recognition. Our own results together with Franklin and Adams (2010) suggest that to the degree that the variance unaccounted for by verbal IQ reflects implicit social-perceptual processes, these processes did not contribute to differences in a more basic face-processing task.

The Eyes Test was originally developed for the study of high-functioning individuals on the autism spectrum as well as their family members (e.g., Baron-Cohen and Hammer, 1997; Baron-Cohen et al., 1997). Since these early studies, the instrument has proved valuable for the examination of individual differences among normally developing samples (e.g., Baron-Cohen et al., 2001). Given both the notion of a broader autism phenotype (Baron-Cohen and Hammer, 1997) and the understanding that autism symptomatology is normally distributed in the general population (Constantino and Todd, 2003), we were motivated to explore the degree to which any influence of verbal IQ might overlap with either phonological memory or figurative language, as has been observed both in individuals with autism and in the broader phenotype. We were particularly interested in the relationship between figurative language and Eyes Test performance, given that this aspect of language processing is specifically related to social cognition. Indeed, in the cases of both autism, schizophrenia, and bipolar disorder this particular language deficit have been related to degree of social-cognitive impairment (McClure et al., 2005; Losh and Piven, 2007; Gavilan and Garcia-Albea, 2011). In our results, phonological memory did not correlate with Eyes Test performance. We did obtain a correlation between the measure of figurative language ability and Eyes Test performance. However, the follow-up regression analysis determined that this effect is not independent from verbal IQ. The results of our study are consistent with the hypothesis that while these specific linguistic measures may explain variance in a disordered population or within a sample of individuals who possess a subset of the genetic risk factors for an autism spectrum disorder, they do not help to explain individual differences within a normal population.

Our study included a number of limitations that should be considered while interpreting our results. First, it must be emphasized that we can only speculate about the precise processes involved in the Eyes Test and the role of verbal IQ in contributing to performance differences. While researchers agree that mentalizing in natural contexts must involve many separate processes that range from implicit perceptual to rich conceptual processing (e.g., Frith and Frith, 2008), it is not clear which processes mediate performance in this measure. Further, fundamental questions remain as to the neurocognitive basis of theory of mind and the degree to which explicit and implicit processes involve discreet or overlapping mechanisms (e.g., Evans, 2008; Apperly and Butterfill, 2009). Baron-Cohen et al. (2001) argued in favor of a relatively implicit underlying process. Clearly, a test of individual differences in social cognition would have more value to the degree that performance reflects an implicit mentalizing process rather than a more general cognitive ability involving verbal intelligence. Although our findings cannot provide further insight into the specific role of verbal IQ during task performance, they raise questions about the degree to which the task is relatively free of general cognitive ability. It should be noted that to the degree that individuals employ different strategies on the task, the relative contributions of implicit and explicit processes may vary across individuals. Unfortunately, we cannot tease apart process differences within individuals.

In more than 250 studies across a wide range of research contexts, the Eyes Test has provided an index of individual differences in mental state understanding (Kirkland et a., under review). While we recognize the productivity of this instrument, we believe our findings highlight the value of further investigation both to guide interpretation in studies involving this instrument and toward the broader goal of understanding the neurocognitive basis of mental state understanding.

## Conflict of Interest Statement

The authors declare that the research was conducted in the absence of any commercial or financial relationships that could be construed as a potential conflict of interest.
